# Opioid Administration and Prescribing in Older Adults in U.S. Emergency Departments (2005–2015)

**DOI:** 10.5811/westjem.2018.5.37853

**Published:** 2018-06-11

**Authors:** Erin M. Marra, Maryann Mazer-Amirshahi, Peter Mullins, Jesse M. Pines

**Affiliations:** * Aventura Hospital and Medical Center, Department of Emergency Medicine, Aventura, Florida; † MedStar Washington Hospital Center, Department of Emergency Medicine, Washington, District of Columbia; ‡ The George Washington University, School of Medicine and Health Sciences, Washington, District of Columbia; § The George Washington University, Department of Emergency Medicine, Washington, District of Columbia

## Abstract

**Introduction:**

We assess trends in opioid administration and prescribing from 2005–2015 in older adults in United States (U.S.) emergency departments (ED).

**Methods:**

We analyzed data from the National Hospital Ambulatory Medical Care Survey (NHAMCS) survey from 2005 to 2015. ED visits for painful conditions were selected and stratified by age (18–64, 65–74, 75–84, ≥ 85 years). We analyzed trends in opioid administration in the ED and prescribing at discharge to encounters ≥ 65 and assessed predictors of use using survey-weighted chi-square tests and logistic regression. Trends in the use of five commonly prescribed opioids were also explored.

**Results:**

Opioid administration in the ED and prescribing at discharge for encounters with patients ≥ 65 years fell overall, but not significantly. By contrast, opioid administration in the ED and prescribing at discharge significantly declined for adult encounters 18–64 by 20% and 32%, respectively. A similar proportion of adult encounters ≥ 65 were administered opioids in the ED as 18–64, but adult encounters ≥ 85 had the lowest rates of administration. A smaller proportion of adult encounters ≥ 65 years with painful conditions were prescribed opioids at discharge compared to <65. However, this age-related disparity in prescribing narrowed over the study period. There were shifts in the specific types of opioids administered and prescribed in adult encounters ≥ 65 years over the study period, with the most notable being a 76% increase in hydromorphone administration comparing 2005–06 to 2014–15.

**Conclusion:**

From 2005–15, 1 in 4 to 1 in 10 ED patients with painful conditions were administered or prescribed an opioid in U.S. EDs. Opioids prescribing increased from 2005–11 and then declined from 2012–15, more so among visits in the 18–64 age group compared to ≥ 65 years. Opioid administrating demonstrated a gradual rise and decline in all adult age groups. Age consistently appears to be an important consideration, where opioid prescribing declines with advancing age. Given the nationwide opioid crisis, ED providers should remain vigilant in limiting opioids, particularly in older adults who are at higher risk for adverse effects.

## INTRODUCTION

Debate continues about the use of opioids in older adults. This conversation is complicated by the significant knowledge gaps regarding the safety and efficacy of opioids, factors that predict positive or negative treatment outcomes, approaches to minimize adverse effects of opioids in older adults, and concerns of addiction and misuse.^1^ However, pain is a common symptom experienced by older adults: studies have shown that approximately 50% of community-dwelling older adults experience daily pain, which has a negative impact on physical and mental health-related quality of life.^2^,^3^ Poorly controlled pain in the outpatient setting increases the number of falls, decreases mobility, and raises the risk of coronary artery disease and mortality in older adults.^4^,^5^ In an inpatient setting, poorly controlled pain has been shown to lead to longer hospital stays, missed physical therapy sessions, decreased ambulation, and delirium.^6^,^7^ Managing pain in older adults is a challenge, given the need to balance the effectiveness of the medication, adverse drug effects, and the potential for drug-drug and drug-disease interactions. Opioid pain relievers control pain, yet they carry risks including constipation, dry mouth, nausea, vomiting, dizziness, somnolence and pruritus.^8^ These risks are magnified in older adults.^9^

Some recent studies have shown that older adults are less likely than younger patients to receive analgesia in the emergency department (ED) for acutely painful conditions and have advocated for increased opioid administration in this population when presenting with painful conditions. One single-center, retrospective observational study revealed that older adults presenting with moderate to severe pain were significantly less likely than younger adults to receive an opioid in the ED.^10^ Another single-center study revealed that patients ≥ 80 years presenting with an acute fracture in the ED were less likely to be prescribed an opioid upon discharge than patients < 80 years (55% vs. 75%).^11^ A national study revealed that adults ≥ 75 years of age were 14.6% less likely to receive an opioid analgesic for painful conditions in the ED than adults 35–54 years.^12^

These results need to be weighed against current research that has shown large increases in opioid prescribing over the past 15 years to the adult population and adverse health outcomes that may be related to increased opioid prescribing. From 2001 to 2010 the percentage of ED visits in which an opioid was prescribed increased from 20.8% to 31.0%, based on national-level data. This study further noted a 6.6% increase in opioid utilization to adults ≥ 65 during the studied time frame.^13^ The number of opioid prescriptions per 100 people in the United States (U.S.) increased by 35% between 2000 and 2009.^14^ This has been paralleled by increasing rates of opioid addiction, overdoses, and deaths.^15^

Older adults are increasingly visiting U.S. EDs.^16^ When ED visits involve acute or chronic pain, ED providers must balance medication risks and benefits when making decisions on pain-control strategies. No studies have examined recent trends specifically in older adults. In this study, we used a nationally representative sample of U.S. ED visits to assess trends in opioid administration in the ED and prescribing at discharge for acutely painful conditions from 2005–2015 among older adults. We focused on the use of specific analgesics, reasons for visit, demographic and hospital factors, as well as pain severity.

**Population Health Research Capsule**

What do we already know about this issue?

Opioid use has been increasing in the past 15 years for all age groups, and while opioids can control pain in older adults, there is a risk of adverse effects, abuse and addiction in this special population.

What was the research question?

What are the trends in opioid administration in the emergency department (ED) and prescribing at discharge to adults ≥ 65 from 2005–2015?

What was the major finding of the study?

While opioid administration in the ED and prescribing at discharge significantly declined for adults 18–64 from 2005–2015, there was no significant decline for adults ≥ 65. Older adults were also consistently prescribed fewer opioids than their younger counterparts.

How does this improve population health?

There are clear disparities by age group for opioid use. ED providers need to balance the concerns of increasing rates of opioid abuse and misuse in older adult, with the need for adequate pain control in this population.

## METHODS

### Study Design

We conducted this study using data from the National Hospital Ambulatory Medical Care Survey (NHAMCS) from 2005 to 2015. NHAMCS is a multi-stage probabilistic sample collected by the National Center for Health Statistics (NCHS) at the Centers for Disease Control and Prevention (CDC) using an annual survey of hospital-based EDs. Using NHAMCS, it is possible to generate national-level estimates of characteristics of ED visits, including patient-level characteristics, such as demographics, reasons for visit, diagnoses, services provided, and patient disposition, as well as hospital-level information, including the geographic region and teaching status. Because NHAMCS is a de-identified, publicly available data source, this study was exempted from institutional board review.

### Methods and Measurements

We analyzed NHAMCS data from 2005 to 2015. Beginning in 2005, NHAMCS indicates whether a medication was administered in the ED or prescribed at discharge, which is why we used 2005 as the initial time point in analysis. To ensure analyses were consistent with NCHS recommendations that raw sample sizes for subgroup analyses meet or exceed 30 cases, we combined data into two-year blocks for most analyses. Thus, comparisons in this study were between 2005–06 and 2014–15, except where otherwise noted.

Our sample was restricted to patients who presented with a painful reason for visit; all such reasons for visit are included in Appendix A. Subgroup analyses were stratified by age group (18–64, 65–74, 75–84, ≥ 85), sex, race, disposition, geographic region, utilization of hospital resources (imaging, procedures, and blood work) and pain type (chest pain, abdominal pain, back pain, musculoskeletal pain, dental pain, and other pain). Hospital characteristics analyzed included the teaching status of hospital. We also analyzed self-reported pain scores, with pain scores of 8 or higher categorized as severe pain and scores of 7 or lower categorized as non-severe pain.

We also examined medications used during ED visits. Medications were classified generally as opioids; specific drugs are available in Appendix B. We analyzed the use of specific, commonly used opioids, including codeine, hydrocodone, hydromorphone, morphine and oxycodone.

### Data Analysis

To examine trends in medication utilization, we tabulated survey-weighted proportions of visits in which patients received opioids, stratified by subgroup. Differences in proportions by year were tested using survey-weighted chi-square analyses. To compare proportions in the grouped years at the ends of the study period, we used survey-weighted linear combinations of estimates. To investigate factors associated with opioid utilization in patients aged 65 years or older, we constructed a survey-weighted logistic regression model. The regression model was constructed using opioids as the dependent variable. We entered all relevant clinical and demographic variables as independent variables. The odds of receiving opioids were adjusted for age group, sex, race, disposition, region, and type of pain. All analyses were conducted using Stata, version 14 (College Station, TX). P-values of < 0.05 were considered significant.

## RESULTS

### Trends in Opioid Administration in the ED

The NHAMCS database included 21.0 million ED visits for painful conditions in patients ≥18 years old who were administered opioids in 2005–06 and 21.7 million visits in 2014–15. Comparing the beginning of the study period in 2005–06 to the end 2014–15, we found no significant change in opioid administration in the ED in adult encounters 65–74, 75–84 or ≥85 years of age, while noting a significant decline in patients 18–64 (Table 1). In 2005–06, rates of opioid administration were similar for visits with patients <85 years (range 22.9–23.4%) with considerably lower rates in 85+ visits (18.7%); however, by 2014–15 rates were lower in the 18–64 group (18.6%) and were slightly lower in visits by patients aged 65–84, and had remained stable for the 85+ group. When assessed by year in graphical form, opioid administration from 2005–15 demonstrated a rise in use in all age groups from 2005–11 with the steepest rise in visits with patients 85+. This was followed by a decline in all age groups (Figure 1A).

In subgroup analyses of encounters with patients ≥ 65, we found that opioid administration in encounters declined in females from 2005–06 to 2014–15 (absolute decrease of 5.4%, p-value = 0.007), during encounters with headaches (absolute decrease of 10.9%, p-value 0.013) and musculoskeletal pain (absolute decrease of 6.8%, p-value 0.009). There was no change in administration for the other subgroups or by specific types of pain (Table 2).

### Trends in Opioid Prescribing From the ED

The NHAMCS database included 15.0 million ED visits for painful conditions in encounters for patients ≥18 years old who were prescribed opioids at discharge from the ED in 2005–06 and 13.3 million visits in 2014–15. Comparing 2005–06 to 2014–15, we found no significant change in opioid prescribing to adults 65–74 and 75–84 years of age from the ED. There was a significant decline in opioid prescribing to patients aged 18–64 (absolute decrease of 5.8%, p-value 0.001) (Table 1). When we graphed opioid prescribing over time, we noted a rise from 2005–11, followed by a sharp decline in opioid administration for 18–64 year olds, and less so for other age groups (Figure 1B). There was no change in prescribing based on demographic factors or types of pain from 2005–06 to 2014–15 (Table 2).

### Trends in Specific Opioid Administration and Prescribing

From 2005–06 to 2014–15, hydromorphone had a large increase in administration to adults ≥ 65 with an overall relative increase of 75.6% (Table 3). There were insufficient data to determine changes in administration of oxycodone, hydrocodone, or codeine. There were also insufficient data to determine changes in prescribing of hydromorphone, morphine, oxycodone, hydrocodone and codeine.

### Demographic Factors that Predict Opioid Administration and Prescribing

For both administered and prescribed opioids in older adults, there were significant demographic and visit specific factors that influenced usage (Table 4). Younger age, White race, female gender, hospital location in the Midwest, South or West, admission of patient, a high pain score, diagnostic imaging, ED procedures, blood work, and presentation for abdominal pain, back pain and musculoskeletal pain were all associated with opioid administration in the ED. Patients presenting with chest pain and dental pain were less likely to receive an opioid in the ED. Younger age, hospital location in the South or West, a high pain score, use of computed tomography/magnetic resonance imaging, and presentation for back pain and musculoskeletal pain were associated with higher prescribing of opioids at discharge from the ED. Patients who had blood work done in the ED or presented with chest pain were less likely to receive an opioid prescription.

## DISCUSSION

In this nationally representative sample, we demonstrate the rise and fall of opioid administration and prescribing over the decade 2005–15. There were disparities in prescribing by age, where younger patients appear to have greater declines in both opioid prescribing and administration than older patients, particularly after 2011 when evidence of the opioid crisis was emerging in the popular media and in public health circles. However, rates of prescribing appear to be more impacted by older age than administration of opioids in the ED. This may be the case because in the ED, patients – in particular older adults – can be observed closely for adverse reactions. By contrast, people receiving discharged prescriptions cannot be directly observed for adverse effects, which tend to be greater in older adults. Our study also extends prior work that demonstrated a rise in opioid use and administration from 2001–2010.^13^ That study, however, analyzed combined opioid administration and prescribing, as prior to 2005 these were not separate variables in the NHAMCS database. Another study that looked at the National Ambulatory Medical Care Survey (NAMCS), found that opioid prescribing to adults ≥65 in outpatient clinics, more than doubled from 1999–2010.^17^ Another similar study using the NAMCS database found a nine-fold increase in prescriptions from 1995 to 2010.^18^

The reasons for our findings are likely multi-factorial and related to increased awareness of the opioid epidemic and to the implementation of mitigation strategies. The increasing use of prescription drug monitoring programs in the past decade has been associated with decreased opioid prescribing.^19^,^20^,^21^ In the past 10 years the CDC and the American College of Emergency Physicians, as well as several states (New York, Ohio and Washington), have developed prescribing guidelines for opioids.^22^,^23^ These factors along with a national recognition of concerns for over-prescribing and divergence of opioids may be contributing to the decline in opioid prescribing in the general adult population and the stable usage in older adult populations, rather than an increasing trend.

Prior studies have demonstrated disparities in prescribing and administering to older adults in the ED. These studies had suggested that older adults with acute pain received less analgesia and, specifically, fewer opioids than younger patients in the ED and upon discharge.^24^,^25^ Our study indicates that while this may be an issue when prescribing opioids upon discharge, it has not been an issue with regard to administration of opioids in the ED. The noted decreases in administration and prescribing of opioids to adults 18–64, may have contributed to the similar rates in opioid use between age groups. The lack of significant decline in opioid administration and prescribing to older adults suggests that ED practitioners, although limiting the use of opioids in the general adult population, are aware of the importance of adequate pain control in older adults and of recent guidelines endorsing this.

Administration of opioids in the ED may be necessary for older adults presenting with acute pain or uncontrolled chronic pain. The Beers criteria, developed in 1991 and most recently expanded and revised in 2015, and the screening tool of older people’s prescriptions (STOPP) criteria introduced in 2008, provide physicians with lists of medications to avoid or use with caution in older adults. The Beers criteria note meperidine, muscle relaxants, benzodiazepines, tricyclic antidepressants and long-term use of non-cox selective non-steroidal anti-inflammatory drugs (NSAIDs) as medications to avoid in older adults, but does not list opioids as medications to avoid.^26^,^27^ The STOPP criteria similarly recommends the avoidance of NSAIDs, tricyclic antidepressants, and benzodiazepines in older adults, and discusses avoidance of long-term opioid therapy in patients with chronic constipation, falls, dementia and in those with mild pain. The STOPP criteria explain, however, that opioid therapy is justified in patients with moderate to severe pain or in palliative care.^28^

Administering opioids to older adults is not without risks, and these risks need to be carefully considered prior to starting opioid therapies. A recent meta-analysis demonstrated that opioids are associated with an increased risk of developing delirium in adults (odds ratio [OR] [2.5], 95% confidence interval [CI] [1.2–5.2])^29^ Yet it has also been shown that inadequate treatment of pain is related to increased rates of delirium.^30^ Older adults are also frequently on multiple medications that increase their risks of adverse drug events related to interactions with opioids.^31^ Not only are older adults susceptible to drug-drug interactions, but also to drug-disease interactions. A retrospective analysis of national-level data from Germany showed that 72% of patients prescribed an opioid had the potential for drug-disease interactions.^32^ A population-based cohort study from Canada found a 37% greater risk of fractures or soft tissue injury with low-potency opioids and a 43% greater injury risk for high- potency opioids in adults ≥ 65.^33^

It is also important to note that although opioid addiction and abuse rates are low for older adults with no past medical history of substance abuse, prescribers should be aware of the epidemic of drug overdose deaths related to opioids in the U.S. There was a 200% increase in deaths related to drug overdoses involving opioid pain relievers and heroin from 2000 to 2014.^15^ Awareness of the opioid epidemic can likely account for the declines in both opioid administration and prescribing in patients aged 18–64. Judicious prescribing is critical in the older adult population as well. Studies have reported increasing rates of abuse of prescription opioids and worse outcomes following misuse of opioids for older adults compared to younger adults.^34^,^35^ U.S. ED visits for opioid overdose quadrupled between 1993 and 2010, with patients >50 years of age accounting for a 231% increase.^36^ A study evaluating the shifting demographics of patients in opioid treatment facilities in New York from 1996–2012, found the largest increases in utilization of opioid treatment programs were in adults ≥50 and that by 2012, adults 50–59 made up the largest age group in treatment facilities.^37^ Given these risks for misuse and opioid-related injuries, the American Geriatrics Society (AGS) recommends using initial assessment tools such as the Opioid Risk Tool and the Screener and Opioid Assessment for Patients with Pain (SOAPPR) to screen for patients at risk for opioid addiction and misuse.^38^,^39^

Over the studied time period, we found unique differences in demographics, visit and hospital-related factors between patients ≥65 who were administered or prescribed opioids. Race and gender only seemed to be a factor in administration of opioids to older adults in the ED, with women and patients of White race associated with higher rates of administration in the ED. This discrepancy was not seen in prescribing of opioids to older adults from the ED. Discrepancies in opioid prescribing have been noted in prior studies, with White patients and women more likely to receive opioids for painful conditions than other ethnicities or men; however, we did not note this difference in our older adult population.^40^,^41^ Older adults who were admitted to the hospital were more likely to be administered an opioid in the ED, suggesting that patients receiving opioids in the ED may have a higher acuity illness. Older adults with chest pain and dental pain were less likely to be administered and prescribed opioids. This is in keeping with current recommendations for treating acute coronary syndrome, which recommends opioids as second-line therapy for chest pain after the use of nitroglycerin.^42^ Recent guidelines also do not recommend the use of opioids for dental pain.^43^

Specific opioids also deserve further discussion when considering the changing physiology of aging. Although there has been minimal change in overall opioid-prescribing rates to older adults and especially among the oldest old, we demonstrate a shift towards the use of more potent opioids. Hydromorphone had the greatest increase in administration from 2005–2006 to 2014–15 with a relative increase of 76%. Parenteral hydromorphone is 7–11 times more potent than parenteral morphine and eight times more potent orally than the equivalent morphine dose.^44^,^45^ A prospective cohort trial evaluating intravenous (IV) opioids (morphine and hydromorphone) dosing and outcomes in the ED demonstrated that among patients receiving 1 mg of IV hydromorphone, 15% of patients were over-sedated and 4% were noted to be confused.^46^

While we were unable to determine dosing used in the ED, it is recommended that initial doses of opioid therapy for older adults be lower than those employed by a younger population and slow titration should be done in a carefully monitored setting.^47^,^48^ This concept is even more important in patients who have hepatic or renal impairment. Opioids are primarily metabolized by the liver and create several active and inactive metabolites that undergo renal excretion.^49^ Therefore, dose adjustments are required when prescribing opioids to patients with significant hepatic and renal impairment.^50^

Guidelines have been established to aid the physician in choosing appropriate therapeutic regimens. In response to the paucity of literature on pain management in older adults, the AGS published a set of guidelines in 1998 to establish pain evaluation and pharmacological recommendations for older adults. The guidelines emphasize the importance of completing a thorough assessment of pain, determining the effects of pain on activities of daily living and instrumental activities of daily living (i.e., key tasks that enable safe and independent living), optimizing disease management, and frequent reevaluation for improvement, deterioration or complications from treatment.^51^

## LIMITATIONS

Our study has several limitations. First, it is unclear based on the data whether pain medication was indicated or desired by the patient based on the information available. Neither was it possible to assess for any cognitive deficits in the older-adult populations that may have affected opioid administration and prescribing. Additionally, although the data collection procedures were designed to make a sample representation of the population, there may be inaccuracies. However, the consistency of the NHAMCS methodology should protect against major inaccuracies.^52^ Because information in the database was obtained from individual ED visits, it was impossible to obtain longitudinal information on individuals or determine appropriateness of therapy. Data regarding dosing of medications are also not included. Finally, the chosen reasons for visits, although reviewed by four authors, did not include all the reasons for visit for which an opioid may be prescribed.

## CONCLUSION

In conclusion, we demonstrate the rise and fall of opioids in U.S. EDs from 2005–15, where there were clear disparities by age group, more so for prescribing than for administrations. ED providers need to be aware of increasing rates of opioid abuse and misuse in older adults and should use opioids judiciously. ED providers should also be aware of the multiple published guidelines that emphasize the importance of pain control in older adults, a thorough evaluation of painful conditions, low initial dosages of pain medications, careful titration and thorough follow-up. Further research needs to be conducted into the effects of such published guidelines on opioid use in older adults and the rates of divergence, misuse and abuse in this particularly vulnerable population.

## Supplementary Information





## Figures and Tables

**Figure 1A:**
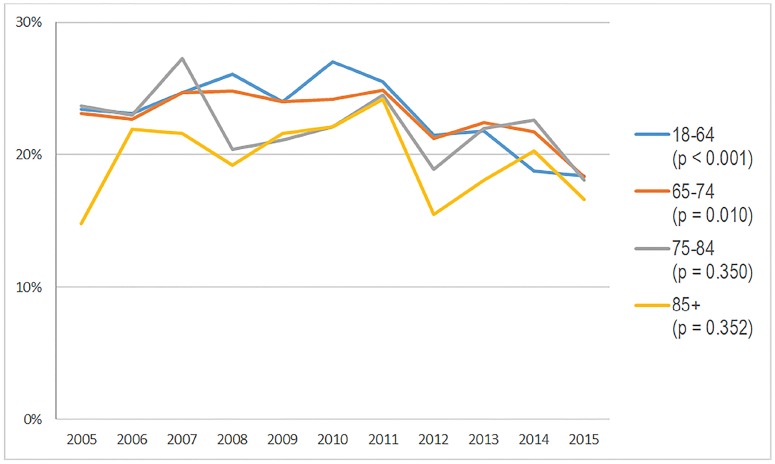
Percentage of patients presenting to United States emergency departments with a painful condition and administered opioids, stratified by age, from the National Hospital Ambulatory Medical Care Survey: 2005–2015.

**Figure 1B:**
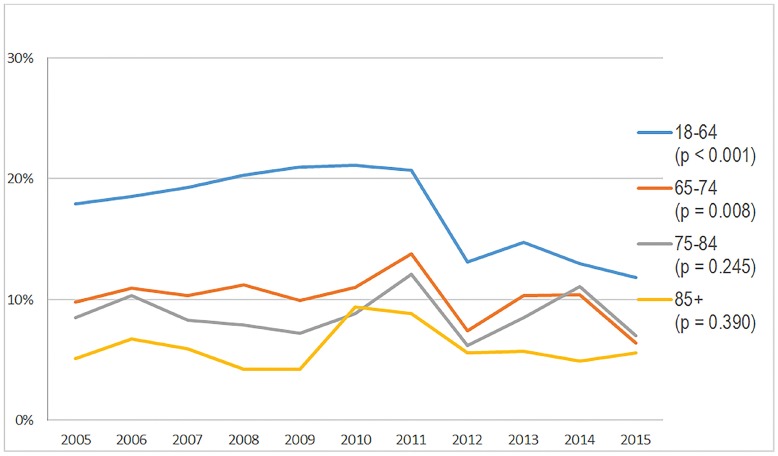
Percentage of patients presenting to United States emergency departments with a painful condition and prescribed opioids, stratified by age, from the National Hospital Ambulatory Medical Care Survey: 2005–2015.

**Table 1:** Percentage of patients presenting to United States emergency departments with a painful condition and administered or prescribed opioids, stratified by age, from the National Hospital Ambulatory Medical Care Survey: 2005–2015.

Age	Estimated number of visits 2005–06	2005–06	95% CI	Estimated number of visits 2014–15	2014–15	95% CI	Relative change	Absolute change	95% CI	P value
Opioids administered in the ED										
18–64	17,113,649	23.3%	(21.9–24.6)	17,191,523	18.6%	(16.7–20.4)	−20.2%	−4.7%	(−6.9–2.4)	0.001*
65–74	2,367,473	22.9%	(20.7–25.0)	2,865,244	20.0%	(17.3–22.6)	−12.7%	−2.9%	(−6.4–0.6)	0.109
75–84	1,051,919	23.4%	(20.3–26.4)	1,119,566	20.3%	(16.6–24.0)	−13.2%	−3.1%	(−7.7–1.7)	0.205
85+	423,000	18.7%	(14.6–22.8)	568,705	18.5%	(13.9–23.2)	−1.1%	−0.2%	(−6.4–6.1)	0.957
Opioids prescribed at discharge										
18–64	13,389,541	18.2%	(17.0–19.4)	11,453,917	12.4%	(11.0–13.8)	−31.9%	−5.8%	(−7.8–3.9)	0.001*
65–74	1,073,687	10.4%	(8.8–12.0)	1,203,855	8.4%	(6.1–10.7)	−19.2%	−2.0%	(−4.7–0.8)	0.161
75–84	422,774	9.4%	(7.4–11.4)	500,111	9.1%	(6.2–12.0)	−3.2%	−0.3%	(−3.9–3.3)	0.869
85+	135,660	-	-	159,490	-	-	-	-	-	-

(-) = Insufficient data, (*) = Significant finding.

*CI*, confidence interval; *ED*, emergency department.

**Table 2:** Percentage of patients ≥ 65, presenting to United States emergency departments with a painful condition and administered or prescribed opioids, stratified by demographic factors and pain type, from the National Hospital Ambulatory Medical Care Survey: 2005–2015.

	2005–06	95% CI	2014–15	95% CI	Relative change	Absolute change	95% CI	P value
Opioids administered in the ED								
Male	19.7%	(16.9–22.5)	21.8%	(17.6–26.1)	10.7%	2.1%	(−2.9–7.2)	0.408
Female	23.7%	(21.2–26.1)	18.3%	(15.6–21.1)	−22.8%	−5.4%	(−9.2–−1.5)	0.007*
White	22.6%	(20.5–24.7)	19.6%	(16.7–22.4)	−13.3%	−3.0%	(−6.7–0.6)	0.105
Non-white	19.9%	(15.5–24.2)	20.7%	(16.5–24.9)	4.0%	0.8%	(−5.4–7.0)	0.798
Admitted	28.1%	(24.7–31.4)	30.7%	(25.3–36.2)	9.3%	2.6%	(−3.9–9.2)	0.426
Discharged	19.6%	(17.2–21.9)	16.7%	(14.2–19.2)	−14.8%	−2.9%	(−6.4–0.7)	0.111
Northeast	17.7%	(15.0–20.4)	14.1%	(9.2–19.1)	−20.3%	−3.6%	(−9.2–2.1)	0.213
Midwest	25.1%	(20.1–30.0)	22.6%	(18.5–26.7)	−10.0%	−2.5%	(−9.2–4.3)	0.471
South	22.4%	(19.2–25.6)	21.0%	(16.0–26.1)	−6.3%	−1.4%	(−7.5–4.6)	0.644
West	23.2%	(18.7–27.6)	18.5%	(14.9–22.2)	−20.3%	−4.7%	(−10.4–1.1)	0.113
Chest pain	14.5%	(11.6–17.4)	14.3%	(10.4–18.3)	−1.4%	−0.2%	(−5.2–4.8)	0.944
Abdominal pain	30.3%	(25.8–34.7)	27.9%	(23.5–32.3)	−7.9%	−2.4%	(−8.5–3.8)	0.45
Back pain	29.3%	(23.9–34.8)	27.9%	(20.3–35.4)	−4.8%	−1.4%	(−11.0–8.0)	0.756
Headache	22.5%	(16.0–29.0)	11.6%	(6.7–16.4)	−48.4%	−10.9%	(−19.6–2.3)	0.013*
Musculoskeletal pain	25.8%	(22.3–29.2)	19.0%	(15.3–22.8)	−26.4%	−6.8%	(−11.8–1.7)	0.009*
Tooth/mouth pain	-	-	-	-	-	-	-	-
Opioids prescribed at discharge								
Male	9.5%	(7.5–11.6)	8.4%	(5.5–11.3)	−11.6%	−1.1%	(−4.9–2.6)	0.554
Female	9.6%	(7.9–11.3)	7.5%	(5.4–9.5)	−21.9%	−2.1%	(−4.8–0.4)	0.102
White	9.8%	(8.3–11.3)	7.7%	(5.4–10.0)	−21.4%	−2.1%	(−4.9–0.7)	0.146
Non-white	8.6%	(5.1–12.1)	8.4%	(5.0–11.7)	−2.3%	−0.2%	(−4.7–4.3)	0.924
Northeast	-	-	-	-	-	-	-	-
Midwest	8.3%	(6.2–10.4)	9.3%	(3.7–15.0)	12.0%	1.0%	(−5.1–7.2)	0.738
South	11.9%	(8.7–15.1)	8.9%	(6.0–11.9)	−25.2%	−3.0%	(−7.2–1.3)	0.17
West	9.4%	(7.2–11.7)	8.0%	(5.2–10.7)	−14.9%	−1.4%	(−5.1–2.1)	0.422
Chest pain	-	-	-	-	-	-	-	-
Abdominal pain	6.9%	(4.5–9.4)	4.6%	(2.5–6.8)	−33.3%	−2.3%	(−5.5–0.9)	0.164
Back pain	17.9%	(12.6–23.2)	16.5%	(9.3–23.6)	−7.8%	−1.4%	(−10.6–7.7)	0.758
Headache	-	-	-	-	-	-	-	-
Musculoskeletal pain	13.7%	(11.2–16.1)	9.8%	(6.8–12.9)	−28.5%	−3.9%	(−7.7–0.0)	0.053
Tooth/mouth pain	-	-	-	-	-	-	-	-

(-) = Insufficient data, (*) = Significant finding.

*CI*, confidence interval; *ED*, emergency department.

**Table 3:** Specific opioid administration and prescribing rates to adults ≥ 65 presenting to United States emergency departments with pain, from the National Hospital Ambulatory Medical Care Survey: 2005–2015.

	2005–06	95% CI	2014–15	95% CI	Relative change	Absolute change	95% CI	P value
Opioids administered in the ED								
Hydrocodone	3.0%	(2.3–3.8)	2.7%	(1.9–3.5)	−10.0%	−0.3%	(−1.5–0.8)	0.556
Hydromorphone	4.5%	(3.4–5.6)	7.9%	(6.2–9.6)	75.6%	3.4%	(1.4–5.4)	0.001*
Morphine	10.0%	(8.5–11.5)	9.3%	(7.7–10.9)	−7.0%	−0.7%	(−3.0–1.6)	0.549
Any opioids	22.1%	(20.2–24.1)	19.7%	(17.2–22.2)	−10.9%	−2.4%	(−5.7–0.9)	0.151
Opioids prescribed at discharge								
Hydrocodone	6.9%	(5.6–8.2)	6.3%	(4.8–7.8)	−8.7%	−0.6%	(−2.5–1.4)	0.564
Any opioids	9.6%	(8.2–11.0)	7.8%	(5.8–9.8)	−18.8%	−1.8%	(−4.2–0.7)	0.157

(-) = Insufficient data, (*) = Significant finding.

*CI*, confidence interval; *ED*, emergency department.

**Table 4:** Characteristics of opioid administration and prescribing to patients ≥ 65 presenting to United States emergency departments with pain, from the National Hospital Ambulatory Medical Care Survey: 2005–2015.

	Administered in ED N=18,415,097			Prescribed at discharge N=7,718,120		
		
Total estimated number of visits	AOR	95% CI	P value	AOR	95% CI	P value
Age						
65–74	Ref	Ref	Ref	Ref	Ref	Ref
75–84	0.82	(0.73–0.92)	0.001*	0.72	(0.60–0.85)	0.001*
85+	0.66	(0.57–0.76)	0.001*	0.49	(0.39–0.62)	0.001*
Race						
Non-white	Ref	Ref	Ref	Ref	Ref	Ref
White	1.25	(1.06–1.49)	0.009*	1.01	(0.80–1.28)	0.905
Sex						
Male	Ref	Ref	Ref	Ref	Ref	Ref
Female	1.2	(1.07–1.35)	0.003*	0.94	(0.79–1.11)	0.441
Region						
Northeast	Ref	Ref	Ref	Ref	Ref	Ref
Midwest	1.81	(1.42–2.29)	0.001*	1.05	(0.80–1.39)	0.707
South	1.36	(1.09–1.69)	0.006*	1.41	(1.05–1.90)	0.022*
West	1.48	(1.18–1.86)	0.001*	1.48	(1.11–1.97)	0.007*
Admitted						
No	Ref	Ref	Ref			
Yes	1.64	(1.41–1.90)	0.001*			
Teaching status of hospital						
Teaching	Ref	Ref	Ref	Ref	Ref	Ref
Non-teaching	1.03	(0.83–1.27)	0.81	1.22	(0.94–1.57)	0.136
Severe pain (Pain score ≥8)						
No	Ref	Ref	Ref	Ref	Ref	Ref
Yes	2.8	(2.50–3.14)	0.001*	1.75	(1.50–2.04)	0.001*
Imaging						
CT/MRI	1.73	(1.55–1.94)	0.001*	1.4	(1.17–1.68)	0.001*
X-ray	1.19	(1.06–1.34)	0.004*	0.99	(0.85–1.15)	0.887
Ultrasound	1.34	(1.01–1.78)	0.045*	1.02	(0.73–1.41)	0.926
Procedure in ED						
No	Ref	Ref	Ref	Ref	Ref	Ref
Yes	1.85	(1.62–2.13)	0.001*	0.93	(0.78–1.11)	0.412
Blood work						
No	Ref	Ref	Ref	Ref	Ref	Ref
Yes	1.25	(1.09–1.42)	0.001*	0.53	(0.44–0.63)	0.001*
Painful condition						
Other pain	Ref	Ref	Ref	Ref	Ref	Ref
Chest pain	0.62	(0.52–0.75)	0.001*	0.32	(0.23–0.45)	0.001*
Abdominal pain	1.29	(1.08–1.55)	0.006*	0.81	(0.63–1.03)	0.082
Back pain	1.74	(1.46–2.08)	0.001*	1.42	(1.15–1.77)	0.001*
Headache	0.87	(0.66–1.13)	0.29	0.7	(0.48–1.03)	0.07
Musculoskeletal pain	1.47	(1.25–1.73)	0.001*	1.28	(1.04–1.56)	0.017*
Tooth/mouth pain	0.47	(0.23–0.94)	0.033*	0.8	(0.43–1.48)	0.472

* = significant finding.

*CI*, confidence interval; *ED*, emergency department; *AOR*, adjusted odds ratio; *Ref*, reference; *MRI*, Magnetic resonance imaging; *CT*, Computed tomography.

## References

[1] Reid MC, Bennett DA, Chen WG (2011). Improving the pharmacologic management of pain in older adults: identifying the research gaps and methods to address them. Pain Med.

[2] Sawyer P, Bodner EV, Ritchie CS (2006). Pain and pain medication use in community-dwelling older adults. Am J Geriatr Pharmacother.

[3] Lapane KL, Quilliam BJ, Benson CW (2015). Impact of non-cancer pain on health-related quality of life. Pain Pract.

[4] Leveille SG, Jones RN, Kiely DK (2009). Chronic musculoskeletal pain and the occurrence of falls in an older population. JAMA.

[5] Zhu K, Devine A, Dick IM (2007). Association of back pain frequency with mortality, coronary heart events, mobility and quality of life in elderly women. Spine (Phila Pa 1976).

[6] Morrison RS, Magaziner J, McLaughlin MA (2003). The impact of post-operative pain on outcomes following hip fracture. Pain.

[7] Lynch E, Lazor M, Gelis J (1998). The impact of postoperative pain on the development of postoperative delirium. Anesth Analg.

[8] Labianca R, Sarzi-Puttini P, Zuccaro SM (2012). Adverse effects associated with non-opioid and opioid treatment in patients with chronic pain. Clin Drug Investig.

[9] Moore RA, McQuay HJ (2005). Prevalence of opioid adverse events in chronic non-malignant pain: systematic review of randomized trials of oral opioids. Arthritis Res Ther.

[10] Hwang U, Richardson LD, Harris B (2010). The quality of emergency department pain care for older adult patients. J Am Geriatr Soc.

[11] Terrell KM, Hui SL, Castelluccio P (2010). Analgesic prescribing for patients who are discharged from an emergency department. Pain Med.

[12] Platts-Mills TF, Esserman DA, Brown L (2012). Older US emergency department patients are less likely to receive pain medication than younger patients: Results from a national survey. Ann Emerg Med.

[13] Mazer-Amirshahi M, Mullins PM, Rasooly I (2014). Rising opioid prescribing in adult U.S. emergency department visits: 2001–2010. Acad Emerg Med.

[14] Kenan K, Mack K, Paulozzi L (2012). Trends in prescriptions for oxycodone and other commonly used opioids in the United States, 2000–2010. Open Med.

[15] Rudd RA, Aleshire N, Zibbell JE (2016). Increases in drug and opioid overdose deaths - United States, 2000–2014. MMWR Morb Mortal Wkly Rep.

[16] Pines JM, Mullins PM, Cooper JK (2013). National trends in emergency department use, care patterns, and quality of care of older adults in the United States. J Am Geriatr Soc.

[17] Steinman MA, Komaiko KDR, Fung KZ (2015). Use of opioids and other analgesics by older adults in the United States, 1999–2010. Pain Medicine.

[18] Olfson M, Wang S, Iza M (2013). National trends in the office based prescription of Schedule II opioids. J Clin Psychiatry.

[19] Suffoletto B, Lynch M, Pacella CB (2018). The effect of a statewide mandatory prescription drug monitoring program on opioid prescribing by emergency medicine providers across 15 hospitals in a single health system. J Pain.

[20] Moyo P, Simoni-Wastila L, Griffen BA (2017). Impact of prescription drug monitoring programs on opioid utilization among medicare beneficiaries in 10 U.S. states. Addiction.

[21] Rutkow L, Chang HY, Daubresse M (2015). Effect of Florida’s prescription drug monitoring program and pill mill laws on opioid prescribing and use. JAMA Intern Med.

[22] Dowell D, Haegerich TM, Chou R (2016). CDC guidelines for prescribing opioids for chronic pain - United States, 2016. MMWR Recomm Rep.

[23] Washington State Department of Health Inter-agency Guidelines for Prescribing Opioids for Pain.

[24] Brown J, Klein E, Lewis C (2003). Emergency department analgesia for fracture pain. Ann Emerg Med.

[25] Quattromani E, Normansell D, Storkan M (2014). Oligoanalgesia in blunt geriatric trauma. J Emerg Med.

[26] Beers MH, Ouslander JG, Rollinger I (1991). Explicit criteria for determining inappropriate medication use in nursing home residents. Arch Intern Med.

[27] American Geriatrics Society 2015 Beers Criteria Expert Panel (2015). American Geriatrics Society 2015 updated Beers criteria for potentially inappropriate medication use in older adults. J Am Geriatr Soc.

[28] Gallagher P, Ryan C, Bryne S (2008). STOPP (Screening Tool Of Older Person’s Prescriptions) and START (Screening Tool to Alert Doctors to Right Treatment) consensus validation. Int J Clin Pharmacol Ther.

[29] Clegg A, Young JB (2011). Which medications to avoid in people at risk of delirium: a systematic review. Age Ageing.

[30] Morrison RS, Magaziner J, Gilbert M (2003). Relationship between pain and opioid analgesics on the development of delirium following hip fracture. J Gerontol A Biol Sci Med Sci.

[31] Budnitz DS, Lovegrove MC, Shehab N (2011). Emergency hospitalizations for adverse drug events in older Americans. N Eng J Med.

[32] Richarz U, Jacobs A, Spina E (2012). How frequently are contraindicated or warned-against combinations of drugs prescribed to patients receiving long-term opioid therapy for chronic pain?. Pharmacoepidemiol Drug Saf.

[33] Buckeridge D, Huang A, Hanley J (2010). Risk of injury associated with opioid use in older adults. J Am Geriatr Soc.

[34] West NA, Dart RC (2016). Prescription opioid exposures and adverse outcomes among older adults. Pharmacoepidemiol Drug Saf.

[35] West NA, Severtson SG, Green JL (2015). Trends in abuse and misuse of prescription opioids among older adults. Drug Alcohol Depend.

[36] Hasegawa K, Espinola JA, Brown DFM (2014). Trends in U.S. emergency department visits for opioid overdose, 1993–2010. Pain Med.

[37] Han B, Polydorou S, Ferris R (2015). Demographic trends of adults in New York City opioid treatment programs- an aging population. Subst Use Misuse.

[38] Webster LR, Webster RM (2005). Predicting aberrant behaviors in opioid-treated patients: preliminary validation of the Opioid Risk Tool. Pain Med.

[39] Butler SF, Fernandez K, Benoit C (2008). Validation of the revised Screener and Opioid Assessment for Patients with Pain (SOAPP-R). J Pain.

[40] Pletcher MJ, Kertesz SG, Kohn MA (2008). Trends in opioid prescribing by race/ethnicity for patients seeking care in U.S. emergency departments. JAMA.

[41] Serdarevic M, Striley CW, Cottler LB (2017). Sex differences in prescription opioid use. Curr Opin Psychiatry.

[42] Amsterdam EA, Wegner NK, Brindis RG (2014). 2014 AHA/ACC Guideline for management of patients with Non-ST-Elevation Acute Coronary Syndromes. Circulation.

[43] American Academy of Emergency Medicine Model ED Pain Treatment Guidelines https://www.aaem.org/UserFiles/PainTreatmentGuidelines-FINAL-2-10-13.pdf.

[44] Knotkova H, Fine PG, Portenoy RK (2009). Opioid rotation: the science and the limitations of the equianalgesic dose table. J Pain Symptom Manage.

[45] Vallner JJ, Stewart JT, Kotzan JA (1981). Pharmacokinetics and bioavailability of hydromorphone following intravenous and oral administration to human subjects. J Clin Pharmacol.

[46] Connor AB, Zwemer FL, Hays DP (2010). Intravenous opioid dosing and outcomes in emergency patients: a prospective cohort analysis. Am J Emerg Med.

[47] Naples JG, Gellad WF, Hanlon JT (2016). The role of opioid analgesics in geriatric pain management. Clin Geriatr Med.

[48] Forman WB (1996). Opioid analgesic drugs in the elderly. Clin Geriatr Med.

[49] Smith HS (2011). The metabolism of opioid agents and the clinical impact of their active metabolites. Clin J Pain.

[50] Smith HS (2009). Opioid Metabolism. Mayo Clin Proc.

[51] AGS Panel on Chronic Pain in Older Persons (1998). The management of chronic pain in older persons. J Am Geriatr Soc.

[52] McCaig LF, Burt CW (2012). Centers for Disease Control and Prevention. Ambulatory health care data. Understanding and interpreting the National Hospital Ambulatory Medical Care Survey: Key questions and answers. Ann Emerg Med.

