# Supplementary material for: Opioid Administration and Prescribing in Older Adults in U.S. Emergency Departments (2005–2015)
Source: West J Emerg Med. 2018 Jun 11;19(4):678–88. doi: 10.5811/westjem.2018.5.37853 (PMC6040900; doi:10.5811/westjem.2018.5.37853)
Supplement: Supplementary file 1 [file wjem-19-678-s001.docx]

**Appendix A:**

| **Painful Conditions (Determined from the Primary Reason for Visit)** | |
| --- | --- |
| Abdominal pain | Lung pain |
| Ankle pain | Migraine |
| Anus/rectum pain | Mouth pain |
| Arm pain | Neck pain |
| Back pain | Pain and related symptoms |
| Bladder pain | Pain during pregnancy |
| Bone pain | Pain, unspecified |
| Breast pain | Painful menstruation |
| Burning sensation | Painful urination |
| Chest discomfort | Pelvic pain |
| Chest pain | Penis pain |
| Chest pain, soreness | Postpartum pain |
| Earache | Scrotum/testes pain |
| Elbow pain | Shoulder pain |
| Foot and toe pain | Sinus pain |
| Gum pain | Skin pain |
| Hand and finger pain | Temporomandibular joint pain |
| Headache | Throat pain |
| Hip pain | Tongue pain |
| Kidney pain | Toothache |
| Knee pain | Unspecified joint pain |
| Leg pain | Unspecified muscle pain |
| Liver/gallbladder/biliary tract pain | Vaginal pain |
| Low back pain | Wrist pain |
