# Supplementary material for: Opioid Administration and Prescribing in Older Adults in U.S. Emergency Departments (2005–2015)
Source: West J Emerg Med. 2018 Jun 11;19(4):678–88. doi: 10.5811/westjem.2018.5.37853 (PMC6040900; doi:10.5811/westjem.2018.5.37853)
Supplement: Supplementary file 2 [file wjem-19-678-s002.docx]

**Appendix B: Classification of Opioids**

| **Schedule II Analgesics** | |
| --- | --- |
| Acetaminophen with Oxycodone | Nucynta |
| Alfenta | Numorphan |
| Alfentanil | Opana |
| Astramorph | Opana ER |
| Atiq | Opioids |
| Avinza | Oramorph |
| B & O Suppository | Oxy IR |
| Belladonna and Opium | Oxycet |
| Demerol | Oxycodone and acetaminophen |
| Dilaudid | Oxycodone and APAP |
| Dilaudid with Phenergan | Oxycodone CR |
| Dolophine | Oxycodone ER |
| Duragesic | Oxycodone HCL |
| Duramorph | OxyContin |
| Endocet | OxyFast |
| Fentanyl | OxyIR |
| Fentanyl Citrate | Percocet |
| Fentora | Percocet 10 |
| Kadian | Percocet 7.5 |
| Magnacet | Remifentanil |
| Mepergan | Roxanol |
| Meperidine | Roxicet |
| Meperidine HCL | Roxicodone |
| Meprozine | Roxicodone Intensol |
| Methadone | Roxilox |
| Morphine | Sublimaze |
| MS Contin | Sufentanil |
| MSIR | Suftenta |
| Narcotic Analgesic | Tylox |
| Neo-Fentanyl |  |

| **Schedule III-V Analgesics** | |
| --- | --- |
| Acetaminophen with Codeine | Nalbuphine |
| APAP with Codeine | Norco |
| Aspirin with Codeine | Nubain |
| Bufferin with Codeine | Pentazocine |
| Buprenex | Phenaphen with Codeine |
| Buprenorphine | Propo-N |
| Butorphanol | Propofan |
| Capital with Codeine | Propoxyphene |
| Codeine | Stadol |
| Codeine Phosphate | T-Gesic |
| Codeine Sulfate | Talacen |
| Fioricet with Codeine | Talwin |
| Fiorinal #3 | Tylenol #2 |
| Fiorinal with Codeine | Tylenol #3 |
| Hydrocodone GF | Tylenol #4 |
| Hydrocodone with Acetaminophen | Tylenol with Codeine |
| Hydrocodone with APAP | Tylenol with Codeine Elixir |
| Hydrocodone with APAP | Vicodin |
| Ibudone | Vicodin ES |
| Lorcet | Vicodin HP |
| Lorcet Plus | Vicoprofen |
| Lorcet HD | Xodol |
| Lortab |  |
